# Antibiotics in agriculture and the risk to human health: how worried should we be?

**DOI:** 10.1111/eva.12185

**Published:** 2014-08-02

**Authors:** Qiuzhi Chang, Weike Wang, Gili Regev-Yochay, Marc Lipsitch, William P Hanage

**Affiliations:** 1 Department of Epidemiology, Harvard School of Public HealthBoston, MA, USA; 2 Infectious Disease Unit, Sheba Medical CenterRamat-Gan, Israel; 3 The Sackler School of Medicine, Tel-Aviv UniversityTel-Aviv, Israel

**Keywords:** agriculture, antibiotic resistance, *Enterococcus*, food, resistance transfer, *Staphylococcus aureus*

## Abstract

The use of antibiotics in agriculture is routinely described as a major contributor to the clinical problem of resistant disease in human medicine. While a link is plausible, there are no data conclusively showing the magnitude of the threat emerging from agriculture. Here, we define the potential mechanisms by which agricultural antibiotic use could lead to human disease and use case studies to critically assess the potential risk from each. The three mechanisms considered are as follows 1: direct infection with resistant bacteria from an animal source, 2: breaches in the species barrier followed by sustained transmission in humans of resistant strains arising in livestock, and 3: transfer of resistance genes from agriculture into human pathogens. Of these, mechanism 1 is the most readily estimated, while significant is small in comparison with the overall burden of resistant disease. Several cases of mechanism 2 are known, and we discuss the likely livestock origins of resistant clones of *Staphylococcus aureus* and *Enterococcus faecium*, but while it is easy to show relatedness the direction of transmission is hard to assess in robust fashion. More difficult yet to study is the contribution of mechanism 3, which may be the most important of all.

The significance of agricultural antibiotics in the emergence and spread of clinical antibiotic resistance is a matter of ongoing debate and controversy, with one prominent commentary asserting (albeit without support or citation) that ‘farming practices are largely to blame for the rise of antibiotic-resistant strains’ (Kennedy 2013). Antibiotic-resistant infections have been conservatively estimated to cause some 23 000 deaths each year in the USA alone (Centers for Disease Control and Prevention 2013) and have been described in apocalyptic terms by public health authorities. If agriculture is a contributor to the spread of resistance, immediate action is necessary to limit this source of human, as well as animal, morbidity, and mortality. However, as we shall argue, the magnitude of the threat arising from the agricultural setting is uncertain for multiple reasons.

Antibiotic use in humans has been shown to select antibiotic-resistant strains, and the same should be expected in livestock, which have been reported to receive over 13 million kilograms, or approximately 80% of all antibiotics, in the USA annually (Hollis and Ahmed 2013). Much of this is not in veterinary medicine, but in the form of continuous subtherapeutic application of antibiotics for growth promotion and disease prevention in intensively farmed animals (Mellon et al. 2001). Unsurprisingly, antibiotics used in this context have been associated with a high frequency of resistant bacteria in the gut flora of chickens, swine, and other food-producing animals (Witte 1998; Aarestrup 1999). Without appropriate regulation, it is thought that a large diverse reservoir for resistant bacteria and resistance genes could facilitate the emergence and spread of resistant pathogens to humans, and even the ongoing transmission of such resistant organisms within the human population.

The maintenance and increase in the prevalence of drug-resistant organisms and resistance genes have been linked to the selective pressure of antibiotic use in both clinical and agricultural settings (Levy 1997). How we should expect drug use in different environments to affect the trajectory of resistant organisms depends on the resulting selective pressure and on constraints to microbial adaptation. Humans are at risk for exposure to new resistant pathogens or resistance determinants from animals through direct contact, ingestion of contaminated food or water, and contact with infected humans. A schematic representation of the possible links between the agricultural setting and human populations is shown in Fig.1. The major influence on the increase in resistant organisms transmitted within each environment is expected to be antibiotic use in that environment. However, we can also consider at least three mechanisms by which antibiotic resistance in agriculture can lead to a threat to humans (Lipsitch et al. 2002).

**Figure 1:**
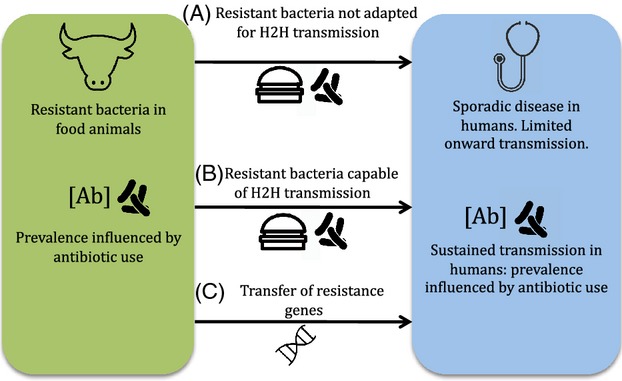
Schematic illustration of possible links between antibiotic use in agriculture and human disease. The prevalence of resistant bacteria in agriculture is influenced by antibiotic use in that setting. The impact of infection depends crucially on the capacity for sustained human to human (H2H) transmission. Arrows linking the two populations represent: a) direct transmission of bacteria not adapted to transmission in humans via the food chain (e.g *Campylobacter*, *Salmonella*) or direct contact with animals; b) direct transmission of organisms already adapted to transmission in humans; c) transfer of resistance genes from the agricultural setting into pathogens transmitting among humans.

1. A human is infected by a resistant pathogen of agricultural origin through contact with livestock, or through ingestion of bacteria from contaminated meat or water, without ongoing transmission of the pathogen between humans.
2. A human is infected or colonized with a resistant microbe through any of these means, followed by ongoing transmission among humans, with some of these humans becoming ill. This scenario constitutes a break in the ‘species barrier’ by a microbe that may be directly pathogenic to humans or may be a commensal with the ability to cause opportunistic infections.
3. Resistance genes arising in the agricultural setting are introduced into human pathogens by horizontal gene transfer. The resulting resistant lineages are then selected by antibiotic use in humans.

Risk modeling may be used to estimate the burden of disease resulting from mechanism 1, although to date it has largely been restricted to infection through food. In the case of direct infection of humans (mechanisms 1 and 2), whether or not it is accompanied by onward transmission (mechanism 2), molecular epidemiology can in principle identify a link between agricultural bacteria, and those infecting humans, if appropriate samples are available. However, with the passage of time, this may become more difficult for organisms that have crossed the species barrier (mechanism 2), as lineages in humans and animals diverge from the common ancestor of the strain that crossed the species barrier. In the case of resistance genes (mechanism 3), this is particularly so, while we might be able to state that the genetic elements or mutations causing resistance in different settings are extremely closely related, we will be unable to identify the direction of transfer with confidence. The central question then becomes: How likely are antibiotic-resistant strains to arise in humans from agricultural antibiotic use as a result of bacterial adaptations, and how likely are these resistant strains to be a cause of significant clinical disease? To lend a more concrete perspective on this complex issue, we shall ground our discussion in salient case studies that highlight the dominant pathways through which such antibiotics may impede human health.

## Sporadic human infection from contaminated food or direct contact

Humans and other animals can acquire resistant pathogens and commensal organisms simply by ingesting them. Contaminated meat and other cross-contaminated foods cause millions of cases of gastrointestinal illnesses such as salmonellosis and campylobacteriosis each year in the USA alone (Scallan et al. 2011). The threat that antibiotic use in food-producing animals poses to human health via this route has been estimated using microbial risk assessment models (McEwen 2012). Using an exposure-based model, one study assessed how many cases of *Campylobacter jejuni* infection complicated by fluoroquinolone treatment failure (i.e., resistant cases) could arise from contaminated ground beef. The study estimated 12 cases in the USA after one year of fluoroquinolone use in cattle, rising to 44 cases and one death after 10 years (Anderson et al. 2001). The comparatively small burden estimated by this analysis is in contrast with the results of another model using an outcome-attribution approach, which predicted an 410 926 excess days of illness annually in the USA due to fluoroquinolone-resistant *Campylobacter* infections, attributed to fluoroquinolone use in animals (Travers and Barza 2002). Subsequently, the United States Food and Drug Administration (FDA) conducted an assessment of the human health impact of fluoroquinolone-resistant *Campylobacter* associated with the consumption of chicken, which was later used to support the withdrawal of fluoroquinolone use in poultry in the USA. The model estimated that between 4960 and 14 370 patients in 1998 were prescribed fluoroquinolone for fluoroquinolone-resistant *Campylobacter* infections attributed to chicken (Bartholomew et al. 2003). The inconsistency in these results may arise from differences in the modeling approaches applied, the outcome units used and the genuine uncertainty surrounding these estimates, reflected by broad confidence intervals (IOM 1988; McEwen 2012). However, these estimates make it clear that a direct impact on human health through resistant pathogens acquired from livestock is plausible.

While contaminated food can cause infections in the context of outbreaks, the causative organisms do not typically transmit between humans, and this may limit the impact of resistance. A comprehensive review of publicly available risk assessments in the USA, which have mainly considered human infection via foodborne transmission rather than direct contact, indicated that the majority of models focused on morbidity and quality of life as endpoints rather than mortality. Notably, no risk assessment model has found more than 100 annual deaths in the USA caused by antibiotic use in food animals (McEwen 2012). Among one of the studies that examined mortality, the Institute of Medicine estimated that 40 deaths due to salmonellosis occur each year in the USA due to subtherapeutic use of penicillin and tetracycline, although as in the examples of fluoroquinolone resistance in *C. jejuni* above, considerable uncertainty surrounds this estimate (IOM 1988). While these models can paint a telling picture of the clinical impact of antibiotics use in food-producing animals, a lack of concrete data, especially regarding the prevalence of resistant strains in food-producing animals, make reliable estimates about the impact in human medicine elusive. However, given what is available, the relatively low numbers indicate that antibiotic use in animals may not be as significant a public health problem in terms of direct transmission, as has been alleged by numbers such as CDC's ‘lower bound’ estimate of 23 000 annual deaths in the USA caused by resistant infections (Centers for Disease Control and Prevention 2013). In addition, very few studies have examined the extent to which restrictions of agricultural antibiotics use would reduce the incidence of human antibiotic-resistant infections caused by this particular mechanism. It has been suggested that the benefits of antibiotics in preventing livestock disease result in an overall reduced risk to humans, that is, the withdrawal of antibiotics could lead to an increase in human disease, albeit susceptible (Cox and Popken 2006). This conclusion is dependent on the hypothesis that antibiotic use in animals leads to fewer pathogenic bacteria entering the food chain, as a result of a lower burden of disease in food animals. This study has been criticized on several grounds, including that it overlooks the contribution of commensal-contaminating flora, that it takes no account of individuals at greater risk of disease, and crucially assumes no difference between disease due to sensitive and resistant strains. It is reasonable to suggest that this is not the case due to the difficulty in treating resistant disease and concomitant risk of sequelae (Claycamp 2006). Moreover, the authors of this study are in receipt of considerable industry funding, a potential conflict of interest, and as a result we must note that while superficially plausible this conclusion is reliant upon questionable assumptions and is counter to the overwhelming weight of expert opinion (Marshall and Levy 2011).

## Ongoing transmission in humans of resistant strains originating in livestock

Following exposure of humans-to-resistant organisms from agriculture, there may be further spread within the human population. Varying degrees of onward transmission within humans have been documented for different clonal types of methicillin-resistant *Staphylococcus*, originally acquired from livestock, such as ST398 in the Netherlands, CC93 in Denmark, and ST 130 in Europe (Armand-Lefevre et al. 2005; Harrison et al. 2013; Spoor et al. 2013). ST398 carriage in farmers appears to be transient; although they are readily colonized, ST398 does not spread easily to family members and the community (Graveland et al. 2011). This suggests constraint in terms of clearing the species barrier to transmit efficiently in humans (or possibly a cost to resistance, although it should be noted that this has not impeded the emergence of numerous community acquired MRSA clones from nonlivestock sources). However, several cases of human infections with ST398 without any contact with livestock or pig farms have been reported (Van der Mee-Marquet et al. 2011). It is unclear how these individuals have acquired these strains, whether through human-to-human contact, or through other exposure routes such as contact with pets, contaminated food, or environmental sources. This reflects poor sampling and limited data in the global surveillance of pathogens that colonize both humans and animals (Fan et al. 2009). While surveillance systems based on clinical infections are capable of monitoring occurrences of resistant pathogenic zoonotic bacteria such as *Salmonella* or *Campylobacter* species, zoonotic transfers of commensals including *Staphylococcus aureus* and *Enterococcus* usually go unnoticed. This possibility for animal-to-human transmission, in the case of MRSA ST398, brings heightened concerns about livestock as potential reservoirs of zoonotic infections that may with further evolution become adapted to circulation within the human population.

In general, there is still a marked gap in our knowledge regarding the successful transfer of resistant bacteria from animals to humans, or vice versa. However, it is clear that the species barrier has been breached multiple times in both directions, with human-adapted strains giving rise to animal-adapted lineages as well as vice versa (Weinert et al. 2012; Shepheard et al. 2013; Spoor et al. 2013). In the case of *S. aureus*, population genetic analyses have clearly shown the existence of host-specific clonal lineages, implying that its adaptive evolution has led to host restriction due to ecological differences among different hosts (Fitzgerald 2012) MRSA ST398 derived from animals appears to be a case of mechanism 1 (Fig.1), and uncertainty still remains about its origin and its implications in public health. In contrast, CC97 appears to have contributed two distinct host-switching events from animals to humans, resulting in sustained onward transmission and representing a clear example of mechanism 2 (Spoor et al. 2013).

Another example of mechanism 2 may be vancomycin-resistant enterococci (VRE). The *Enterococcus*, which normally colonizes the gut, has acquired resistance to multiple antibiotics over time, making the glycopeptide vancomycin one of the last therapeutic options. The epidemiology of VRE differs substantially between the USA and Europe. In Europe, *Enterococcus faecium* carrying the *vanA* resistance element for vancomycin resistance was commonly found in the intestinal flora of farm animals as well as healthy people, but carriage of VRE in farm animals and healthy people was absent in the USA until 2008 (Bonten et al. 2001). This difference has been proposed to be due to the widespread agricultural use of avoparcin, a glycopeptide used in Europe since the 1970s, but was never approved for use in the USA. Avoparcin, which confers cross-resistance to vancomycin, has been shown to select for VRE in animals (Aarestrup et al. 1996). A large reservoir of VRE in animals presents many opportunities for human infection, and the potential for resistant bacteria to colonize the human niche. Molecular epidemiologic studies have found that the VRE strains isolated from animals and humans are similar, as are the resistance elements (Woodford et al. 1998; Jensen et al. 1999); hence it is clear that at least the potential for transmission exists.

The combined epidemiological and molecular data provide indirect evidence that vancomycin resistance may have arisen from agriculture and transferred to humans, but the evidence is not conclusive. Notably, although Europe preceded the USA in the circulation of VRE in healthy community-dwellers, the clinical problem of VRE circulating in hospitals emerged first in the USA (Bonten et al. 2001) and was associated with the increase in oral vancomycin use in US hospitals (Handwerger et al. 1993). Furthermore, VRE outbreaks in the USA were mainly due to a particular virulent and adhesive clone, CC17 (Willems et al. 2005). While a recent genomic analysis showed that the CC17 clone was much more closely related to isolates collected from diverse animal sources than a small sample of human commensal isolates (Lebreton et al. 2013), it is not possible to define the direction of transmission without the context of a larger sample. Importantly, none of the samples from the animal population harbored either *vanA* or the alternative resistance gene *vanB*, and so the specific relation of the CC17 clonal complex to agricultural VRE is obscure.

## Horizontal transmission of resistance genes originating from livestock

Bacteria are known to be capable of transferring genes between strains of the same species, and between species, via multiple mechanisms including but not limited to plasmid transfer, phage transduction, and natural transformation. Numerous examples are already known of resistance genes arising in one species, perhaps a relatively innocuous commensal, but then being donated to a different, pathogenic species, with consequences for human health (Coffey et al. 1993; Dowson et al. 1993; Bowler et al. 1994). As a result of such bacterial promiscuity, the most significant role of antibiotic use in agriculture may be in facilitating the emergence of new resistance genes, which can then be transferred into pathogens already adapted to transmission in humans. The argument here is that antibiotic use creates a breeding ground for the accumulation and movement of resistance genes, and it is the existence of this reservoir that poses the most serious threat to public health.

As noted above, resistance elements in VRE found in animals and humans are similar, but it is not clear whether this represents transfer of the whole organism or the genes alone. Given the relatively high rates of recombination in the *Enterococcus*, resistance loci such as *vanA* and *vanB* can be readily transmitted into other lineages or bacterial species, with potentially catastrophic effects (Willems et al. 2011). The conjugative transfer of high-level vancomycin resistance to *S. aureus* has been demonstrated *in vitro* and *in vivo* (Noble et al. 1992). The possibility of such transfer may not imply that when it occurs, it will lead to a large clinical problem. To date, only 13 clinical VRSA isolates have been reported in the USA, all related to intensive vancomycin use (Kos et al. 2012; Limbago et al. 2014). This likely reflects biological constraints: An elegant study has shown that acquiring vancomycin resistance results in a fitness cost to the organism as estimated by growth rate and that this is partially compensated by deletion of the *mecA* gene that confers methicillin resistance (Noto et al. 2008). The resulting return to methicillin susceptibility impedes onward transmission. An even more worrisome case may be the recent emergence of the carbapenem-resistant *Enterobacteriaceae*, with a highly transmissible resistance gene for the New Delhi metallo-beta-lactamase (NDM). This gene was demonstrated to easily transfer from one species to another on different plasmids. Zoonotic transmission of this pathogen from chickens has also been demonstrated (Wang et al. 2012).

The transfer of resistance determinants from animal to human through horizontal gene transfer is tremendously difficult to trace and quantify, and the role of animal reservoirs as the ultimate source of genes contributing to clinical resistance remains to be definitively proven. Putting the problem in perspective, the phenomenon of resistance to naturally occurring antibiotics greatly precedes the development of agriculture. The metallo-beta-lactamases (of which NDM is one especially worrisome example) have an extremely ancient origin, so ancient in fact that no detectable sequence homology remains between different classes of these genes (Hall et al. 2004; Bebrone 2007). Horizontal transfer is thought to have been important in the evolution of these enzymes, but whether that process has been accelerated by the use of antibiotics in agriculture is not known.

## The impact of banning antibiotic use in agriculture

The possibility of clinical resistance against our last line of treatment as a result of antibiotic use in animals has led to large-scale antibiotic bans as precautionary measures. By 2006, the European Union had banned all nonmedicinal antibiotics in animals. Antibiotic regulations have also become more stringent in the USA. In 2005, the emergence of fluoroquinolone-resistant *Campylobacter jejuni* in the clinical setting in conjunction with fluoroquinolone administration in animals prompted the FDA to ban fluoroquinolone use in poultry, although it remains unclear if the dramatic increase of fluoroquinolone-resistant strains was due to fluoroquinolone use in livestock (Engberg et al. 2001; Gupta et al. 2004). Recently in December 2013, the FDA issued a voluntary policy that asks farms to cut routine use of antibiotics and consult veterinarians before use.

The ban in Europe created a large-scale natural experiment in which we could observe the effects of an agricultural antibiotics ban. After the avoparcin ban, some reported that the prevalence of VRE in farm animals rapidly declined (Pantosti et al. 1999; Aarestrup et al. 2001). However, this did not translate directly to a decrease of VRE in humans (Phillips 2007). If the ultimate origin of VRE truly lies in agriculture, then it is plausible that an avoparcin ban in the past could have prevented the emergence of this resistance threat. Under this scenario, however, once the species barrier was breached and ongoing transmission in humans established, reducing the risk of additional animal-to-human transfer of resistant organisms may have little impact on the prevalence of such organisms in humans. Indeed, mathematical modeling studies have argued that the greatest impact of agricultural antibiotic bans would be those for antibiotics causing resistance that is not yet clinically important in humans, precisely to prevent crossing of the species barrier (Lipsitch et al. 2002; Smith et al. 2002).

Even after antibiotic selection pressures have been removed or reduced through a ban, it is not clear that there will be a reduction in the presence of resistant organisms in the environment. In the USA, no decline in the levels of ciprofloxacin resistance has been observed following the ban of fluoroquinolones in chickens (Price et al. 2007; Nannapaneni et al. 2009). While it is possible that insufficient time has elapsed for trends to be detectable, it is also possible that fluoroquinolone-resistant strains may remain in the environment in absence of antibiotic selective pressure (Humphrey et al. 2005). This may be due to a minimal cost of resistance, or co-selection for other adaptive features of the resistant organism. A resistance determinant is inherited along with these other adaptations, and so maintained along with them. An example of such co-selection is loci that confer resistance to heavy metals, which are often found in close linkage with resistance loci (Baker-Austin et al. 2006). Discharge of heavy metals into the environment will then select directly for the heavy metal resistance loci, and indirectly for antibiotic resistance. An example is copper resistance (encoded by the *tcrB* gene in glycopeptide-resistant enterococci isolates from pigs (Hasman and Aarestrup 2002). This genetic linkage was connected to the higher copper exposure in pigs through feed additives in Denmark. Unlike antibiotics, heavy metals are not degraded and can linger in the environment producing a long-term selection pressure (Stepanauskas et al. 2005). While the extent to which co-selection of resistance determinants will affect clinical resistance is unclear, the greatest value in restricting antibiotic use, as is the case in human medicine, may not be in reversing resistance, but in preventing further increases in prevalence and the possibility that the relevant genes find themselves horizontally transferred into yet more pathogens.

## Discussion

The topic of agricultural antibiotic use is complex. As we noted at the start, many believe that agricultural antibiotics have become a critical threat to human health. While the concern is not unwarranted, the extent of the problem may be exaggerated. There is no evidence that agriculture is ‘largely to blame’ for the increase in resistant strains and we should not be distracted from finding adequate ways to ensure appropriate antibiotic use in all settings, the most important of which being clinical medicine.

The debate about agricultural use of antibiotics is further complicated by its relation to other scientific, political, and economic issues. The desire for meat raised without antibiotics is part of a larger consumer movement toward more ‘natural’ and sustainable food sources (Silbergeld et al. 2008; Paarlberg 2010). While controversial in light of a trend toward ‘evidence-based’ policymaking (Pugh 2002; Phillips et al. 2004), this precautionary consumer attitude may be desirable even if quantifiable harms of a particular practice are limited. The most consequential impacts of agricultural antibiotic use, such as possibly leading to the origin of loci conferring high-level resistance, are among the hardest to demonstrate conclusively, yet the absence of evidence does not mean that there is no effect (Marshall and Levy 2011). Pointing to agricultural sources for clinical problems of antimicrobial resistance serves the interests of vendors and prescribers of antibiotics for clinical medicine by implying that they bear a correspondingly lesser share of the blame. On the other hand, the strong economic interests favoring continued use of antibiotics in agriculture have resulted in major funding for studies that have found modest human health burdens from agricultural animal use (http://www.cox-associates.com/health.htm, accessed March 2, 2014), although the potential conflicts of interest are not always reported in the peer-reviewed publications reporting these analyses (Cox and Popken 2006).

While overuse of antibiotics in *any* setting is a matter of concern, it remains important to determine what exactly constitutes ‘overuse.’ It is important that we simultaneously preserve effective antibiotics as long as possible, but also that we continue to deploy them in the service of human and animal health. We could stop using antibiotics altogether, and this would greatly reduce the selective pressure they exert (although given the presence of naturally occurring antibiotics, selective pressure would not be removed entirely) but the consequences for public health would obviously be dire. A more pragmatic scenario is that the use of subtherapeutic amounts of agricultural antibiotics as prophylaxis or in growth promotion be closely scrutinized. Low-dose antibiotics favor the emergence of resistance, and this practice has also been condemned as a cover for poor standards of animal care (The Pew Charitable Trusts 2008).

In recent years, agricultural antibiotics have gained a tremendous amount of attention from the media. In determining how much antibiotic use is too much, we must turn to the things we do not know. From the proportion of antibiotics by weight used in agriculture as opposed to human medicine (Hollis and Ahmed 2013), it does not follow that the majority of selective pressure on human pathogens, let alone the majority of human health impact of antibiotic resistance, results from agricultural uses. To establish such a causal mechanism requires quantifying the relationship between quantity of antibiotics used, selection exerted, and human health impact. We also have limited knowledge of the consumption of antibiotics in different animal species and similarly limited surveillance programs to monitor and trace the emergence of resistance in animals (Perron et al. 2008). The limited data available make it hard to quantify the relationship between antibiotic use in animals and the occurrence of clinical resistance. As we have shown, while there is considerable evidence associating antimicrobial use in agriculture with resistant pathogens in livestock and in the food supply, the evidence for human health risks directly attributable to agricultural antibiotics runs the gamut from speculative to scant. There is an urgent need for better studies that combine quality surveillance with good data on antibiotic usage in agriculture, which is at present hard to come by, and any serious attempt to address this problem will require the agricultural industry to be more forthcoming. In determining whether regulations should be in place, we must weigh the need for scientific evidence of an inherently difficult-to-measure phenomenon against the consequences of inaction. Regulations of antibiotic use in agriculture will likely do the most good if they are in place early enough to prevent the rise of antibiotic-resistant strains. Once these strains have emerged, it might be only a matter of time before they cross the species barrier and adapt to living in humans, at which time there is very little regulation of agriculture can do to prevent their persistence in the clinical setting. The greatest value of reducing agricultural antibiotic use now may be in maintaining a status quo that, while far from ideal, is greatly preferable to the alternative.
